# Two Opposing Functions of Angiotensin-Converting Enzyme (ACE) That Links Hypertension, Dementia, and Aging

**DOI:** 10.3390/ijms222413178

**Published:** 2021-12-07

**Authors:** Duc Le, Lindsay Brown, Kundan Malik, Shin Murakami

**Affiliations:** Department of Basic Sciences, Master of Science in Medical Health Sciences, College of Osteopathic Medicine, Touro University California, Vallejo, CA 94592, USA; dle5@student.touro.edu (D.L.); lbrown25@student.touro.edu (L.B.); kmalik@student.touro.edu (K.M.)

**Keywords:** aging, age-related comorbidities, angiotensin-converting enzyme, amyloid-degrading enzyme, Alzheimer’ s disease, dementia, hypertension, life extension, stress resistance

## Abstract

A 2018 report from the American Heart Association shows that over 103 million American adults have hypertension. The angiotensin-converting enzyme (ACE) (EC 3.4.15.1) is a dipeptidyl carboxylase that, when inhibited, can reduce blood pressure through the renin–angiotensin system. ACE inhibitors are used as a first-line medication to be prescribed to treat hypertension, chronic kidney disease, and heart failure, among others. It has been suggested that ACE inhibitors can alleviate the symptoms in mouse models. Despite the benefits of ACE inhibitors, previous studies also have suggested that genetic variants of the ACE gene are risk factors for Alzheimer’s disease (AD) and other neurological diseases, while other variants are associated with reduced risk of AD. In mice, ACE overexpression in the brain reduces symptoms of the AD model systems. Thus, we find two opposing effects of ACE on health. To clarify the effects, we dissect the functions of ACE as follows: (1) angiotensin-converting enzyme that hydrolyzes angiotensin I to make angiotensin II in the renin–angiotensin system; (2) amyloid-degrading enzyme that hydrolyzes beta-amyloid, reducing amyloid toxicity. The efficacy of the ACE inhibitors is well established in humans, while the knowledge specific to AD remains to be open for further research. We provide an overview of ACE and inhibitors that link a wide variety of age-related comorbidities from hypertension to AD to aging. ACE also serves as an example of the middle-life crisis theory that assumes deleterious events during midlife, leading to age-related later events.

## 1. Introduction

ACE plays a major role in the angiotensin–renin system that regulates blood pressure and salt balance. ACE was first reported as a hypertensin-converting enzyme [1]. In 1958, the term “hypertensin” was revised to “angiotensin” [2]. ACE inhibitors had been initially reported as a medication to treat hypertension [3,4]. The history of angiotensin and ACE have been described elsewhere [5]. The ACE gene encodes a protein with 732 amino acid residues and a molecular weight of 80,073 [6]. Clinically, it is an important target for the treatment of hypertension. Current first-line medications for hypertension include ACE inhibitors, angiotensin receptor blockers (ARBs), beta-blockers, and calcium channel blockers [7].

Primary hypertension, or essential hypertension, is the condition of having high blood pressure. Although no clear cause for primary hypertension is known, a combination of genetics, poor diet, and lack of exercise is thought to play a role. In contrast, secondary hypertension is high blood pressure caused by another medical condition. Diabetes, Cushing’s syndrome, pheochromocytoma, hyperparathyroidism, hypokalemia, or primary hyperaldosteronism are several examples of conditions that may cause secondary hypertension [8]. Secondary hypertension can be diagnosed following a series of screening and lab tests to identify underlying factors and impacted organs [9].

Our recent studies suggested that the ACE gene is associated with a broad range of neurological diseases, including AD, amyotrophic lateral sclerosis, multiple sclerosis, Parkinson’s disease (PD), and schizophrenia [10,11], which is consistent with the findings from other groups [12,13]. ACE inhibitors are well-established medications for hypertension, which is also a major risk of cognitive impairment and dementia [14] and of death by COVID-19 [15]. Here, we overview the underlying mechanisms of ACE relevant to AD and age-related comorbidities. We hope to provide the links between ACE and hypertension, AD, neurological diseases, and aging.

## 2. Mechanism of ACE

**Figure 1:**
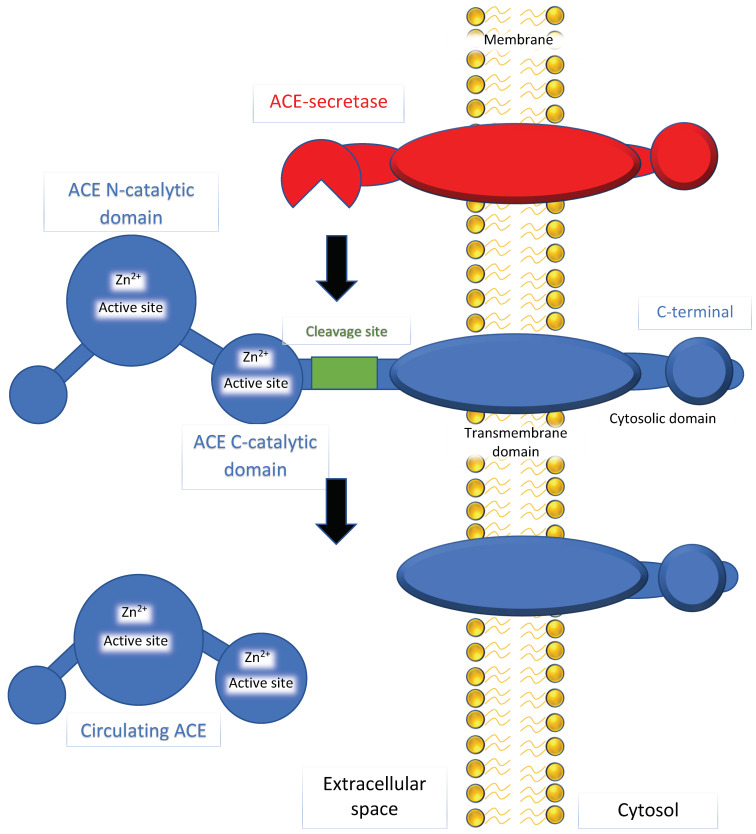
A diagram representing angiotensin-converting enzyme (ACE) and ACE-secretase. Phospholipid bilayer of the cell membrane (indicated by yellow), ACE-secretase bound to the cell membrane (indicated by red), and ACE (indicated by blue). The figures for ACE depict two major domains, the N- and C-catalytic domains, which are active sites that bind zinc ions. ACE-secretase cleaves at a site nearby the N-catalytic domains to form circulating ACE. This circulating and non-circulating ACE can hydrolyze and cleave angiotensin I to form angiotensin II in the mechanisms described in the text.

ACE has a dipeptidyl carboxypeptidase activity that can hydrolyze and cleave the c-terminal dipeptide of angiotensin I (ten amino acid residues) to make angiotensin II (eight amino acid residues). ACE has another activity as an amyloid-degrading enzyme (ADE) that can hydrolyze beta-amyloid (discussed in Section 3.4). As shown in Figure 1, the N and C domains contain active sites with a zinc ion-binding site. Previous studies have reported that inhibition of the C domain can manage blood pressure, while inhibition of the N domain has little or no impact [16,17]. The conversion of angiotensin I to angiotensin II is prevented by binding to the C domain, leading to disruption of the renin–angiotensin–aldosterone system (RAAS). Angiotensin II acts as a vasoconstrictor by stimulating Gq proteins on the vascular smooth muscle, activating an IP3-dependent pathway that increases intracellular calcium levels and causes constriction.

**Figure 2:**
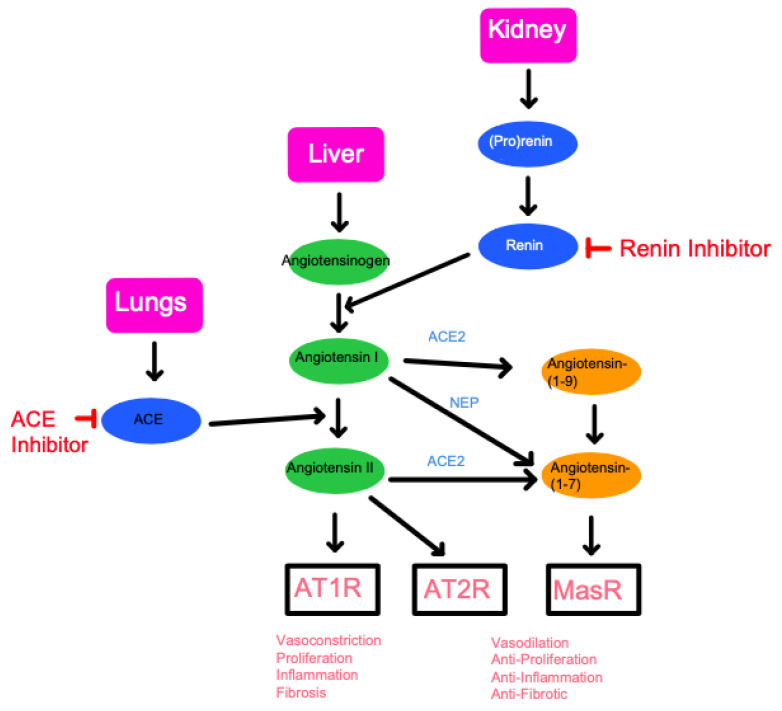
A schematic diagram of the Renin-Angiotensin System (RAS) pathway and the points in which inhibition may occur. The three key organs of the RAS pathway include the lungs, liver, and kidney. The RAS pathway revolves around the production of angiotensinogen, the precursor to angiotensin originating from the liver. Through renin produced in the kidney, followed by ACE produced in the lungs, angiotensin II can subsequently be formed. By inhibiting either renin or ACE, the progress of the RAS pathway can effectively be halted. Below the schematic is a summary of the final physiological effects followed by the binding of each target is listed at the bottom of the diagram. Angiotensin II will produce vasoconstriction, proliferation, inflammation, and fibrosis through binding to the AT1 receptor (AT1R). Binding to AT2 receptor or MasR, following conversion to Angiotensin-(1-7), will result in opposing physiologic effects. This conversion to Angiotensin-(1-7) is known as alternative RAS activation and is enabled by the angiotensin-converting-enzyme-2 (ACE2) and neprilysin (NEP), a protease abundantly found in the kidneys.

Angiotensin II has two major receptors that it can interact with to produce homeostatic actions (Figure 2). Angiotensin II type 1 receptor (AT1R) has well-documented functions in vasoconstriction, cellular proliferation, inflammation, and fibrosis. Due to its significant role in increasing blood pressure, AT1R is a major target for anti-hypertensive drugs, specifically ARBs. Conversely, angiotensin II type 2 receptor (AT2R) does not have as clearly defined of a role as AT1R and is an ongoing subject of research [18]. Past studies have suggested that AT2R primarily functions as an antagonist for AT1R, inducing contradicting effects such as vasodilation and inhibition of cell proliferation, inflammation, and fibrosis. In addition, studies have shown that the Mas receptor, which responds to angiotensin (1-7), induces similar effects to AT2R. Due to this observed function of regulating the proinflammatory effects of angiotensin II, studies have tested and demonstrated the impact of the receptor Mas axis on macrophage function and inflammation diseases [19]. Thus, a defect in the Mas receptors, or a deficiency in ACE2, has been shown to cause an increase in inflammatory responses within the CNS and the vascular systems.

## 3. ACE as a Gene That Links Hypertension, AD, and Aging

### 3.1. ACE as a Link to Hypertension

Hypertension develops with increasing age. Despite varying definitions, a threshold of early-onset hypertension is at the age of fewer than 55 years old [20] when recommending first-line medications, such as ACE inhibitors [21]. ACE generates angiotensin II, which has an effect on vasoconstriction and increases blood pressure. ACE inhibitors can reduce angiotensin II, leading to vasodilation and a subsequent decrease in blood pressure (i.e., vaso-protection against hypertension). ACE inhibitors may also downregulate sympathetic activity, leading to less cardiovascular burden. Other cardiovascular effects of ACE inhibitors include the promoted renal excretion of sodium and water, leading to a further decrease in blood volume. ACE inhibitors and angiotensin II receptor blockers (ARBs) are also known to reduce the risk of hospitalization for heart failure in an ethnicity-dependent manner [22]. Thus, ACE inhibitors and ARBs are first-line medications for treating hypertension, chronic kidney disease, and heart failure.

ACE has been a point of interest in previous studies, which have shown that hypertension is a strong risk factor for developing cognitive impairment and dementia [14]. Abnormalities such as chronically elevated blood pressure or a series of mini strokes have been associated with impaired cognitive function and the onset of various forms of dementia, including AD. A wide variety of cardiovascular diseases are also sensitive to hypertension [23,24].

### 3.2. ACE as a Link to AD and Diverse Neurological Diseases

In humans, ACE polymorphisms are associated with AD. Two groups conducted population studies that suggested an association with late-onset AD (LOAD) by investigating an insertion/deletion polymorphism of the ACE gene [25,26]. However, earlier studies have been controversial: 74 case–control studies and 6 family-based studies have investigated the genetic association between ACE and AD, mixed with 26 positive results, 31 negative results, 16 not applicable, and the remaining being inconclusive, according to the AlzGene database (Accessed 15 November 2021) [27]. A series of meta-analysis studies have been necessary and showed the significance of the genetic association between the ACE gene and AD [10,11,28,29,30,31,32]. More details of ACE gene variations are discussed in the next section.

The ACE gene is associated with a wide range of neurological diseases, including AD, amyotrophic lateral sclerosis (ALS), multiple sclerosis (MS), Parkinson’s disease (PD), and schizophrenia [10,11,12,13]. The neurological diseases have a strong association with ACE, MTHFR, and TNF [10]. The studies have significant clinical implications, namely that patients with a risk factor gene of AD may show diverse neurological symptoms such as ALS, MS, PD, and schizophrenia. Thus, patients with AD may show diverse symptoms in addition to those directly relevant to AD.

The nature of the foundation of the relationship between AD and age-related comorbidities is unclear. However, hypertension and strokes are independent risk factors of cognitive impairment and dementia [14,33]. The onset of cognitive impairment is common after long-term hypertension and stroke [14]. Chronic inflammation is commonly seen in patients with comorbidities including hypertension and diabetes prior to the development of AD [34,35]. Chronic inflammation in the brain may be an intermediary component in the progression of AD and becomes more common with the aging process. The understanding of age-related comorbidities should open a new avenue for research to understand the mechanisms that link a wide variety of pathophysiology in aging.

### 3.3. Genetic Variations of ACE That Link to AD

Genetic variants in ACE have been reported that are associated with the risk of AD, including the insertion/deletion variant and single nucleotide polymorphisms (SNPs). The insertion/deletion variants have an Alu repeat present (insertion) or absent (deletion) [36], which initially showed an association with AD [28,37,38]. More than seven SNPs are associated with AD, including rs4291 [31,32,39,40], rs138190086 [37], rs4343, and rs1800764 [39]. Four variants, rs4968782, rs4459609, rs4316, and rs4343, are associated with decreased risk of AD; rs4316 and rs4343 are synonymous substitutes but are predicted to affect transcription factor binding [41]. It has also been suggested that ACE may also inhibit Aβ aggregation by the amyloid-degrading function [42]. It is also consistent with the implication of clinical studies described below. Interestingly, a rare penetrant ACE variant, rs4980 (R1279Q), seems to cause AD, since the variant can accelerate Aβ-induced neurodegeneration in mice [43] (discussed in Section 3.4). Thus, there are two opposing types of variants: variants associated with increased risk of AD and variants associated with reduced risk of AD.

Clinical studies suggest that ACE polymorphisms affect its level and activity in the cerebrospinal fluid (CSF) and serum, with some inconsistent findings. In previous studies, the insertion/deletion polymorphisms and three SNPs (rs4291, rs4343, and rs1800764) show an increased risk of AD, increased CSF Aβ, and an earlier age of AD onset [39,44]. CSF ACE activity is increased in AD, while CSF ACE levels are reduced. Aβ1-42 can increase the ACE level and activity in the cultured neurons in vitro. Thus, ACE may be induced by Aβ. In another study, of seven SNPs associated with CSF ACE levels, four SNPs (rs4316, rs4343, rs4459609, and rs4968782) are associated with decreased risk of AD and increased ACE levels in CSF and plasma [41]. However, the finding in rs4343 is not consistent with the previous studies showing reduced ACE levels [39,44]. Nonetheless, the clinical studies support the protective function of ACE in the AD pathology, which may involve ACE as an amyloid-degrading enzyme.

### 3.4. Two Opposing Health Effects of ACE on AD

As discussed above, two opposing effects of ACE have been observed. ACE inhibitors can be used as treatment options for hypertension and other health conditions, while alterations of the ACE gene are associated with AD. In addition, there are two types of ACE variants: variants with increased risk of AD, and variants with reduced risk of AD. Why do the seemingly opposing health effects of ACE occur?

We differentiate the effects into two functions:

(1). ACE as an angiotensin-converting enzyme that makes angiotensin II in the renin–angiotensin system. The action of ACE is described in Section 2. ACE inhibitors reduce the production of angiotensin II, which results in vasodilation and reduced blood pressure. In this case, inhibiting the activities as an angiotensin-converting enzyme would relieve hypertension and hypertension-sensitive conditions, including heart failure, chronic kidney disease, or diabetes mellitus [45]. In mice, the role of the ACE/angiotensin II signaling has been investigated. A previous study suggests that a brain-penetrant ACE inhibitor, Captopril, reduces AD symptoms in the mouse AD model (Tg2576) which overexpresses a Swedish APP mutation (KM670/671NL) [46]. In another mouse AD model (5XFAD) that has five AD-linked mutations, ACE variant rs4980 (R1279Q) causes aging-dependent, Aβ-accelerated selective hippocampal neuron vulnerability and female susceptibility [43]. The Aβ-induced hippocampal neurodegeneration is rescued by the ACE inhibitor (Captopril) and AT1R inhibitor/ARB (Losartan) that can penetrate the brain [43]. The studies suggest that ACE/angiotensin II signaling causes Aβ-induced neurodegeneration and that brain-penetrant ACE inhibitor/ARB can protect the neurons. Although clinical studies remain to be completed, it is reasonable to conclude that ACE inhibitors may be beneficial to health under specific conditions.
(2). ACE as an amyloid-degrading enzyme (ADE) that can hydrolyze beta-amyloid and decrease amyloid toxicity. ADE represents a group of broadly defined enzymes, currently, including 14 enzymes: ACE, acyl peptide hydrolase, aminopeptidase A, cathepsin B, endothelin-converting enzyme, glutamate carboxypeptidase II, insulin-degrading enzyme, MBP, MMP-2, MMP-9, NEP2, neprilysin, plasmin, and PreP [47,48]. ACE has an activity of beta-amyloid amyloid-degrading enzymes (ADEs) that can hydrolyze and convert Aβ1-42 to Aβ1-40 in homogenates of the mouse Tg2576 AD model and human AD autopsy [47], which is consistent with its dipeptidyl carboxypeptidase activity that cleaves the c-terminal two amino acid residues. ACE can also cleave Aβ1-40 into two smaller peptides (Aβ1-7 and Aβ8-40) [49,50,51]. In mice, inhibitions of ACE can worsen the accumulation of Aβ1-42 in the mouse model [47]. Consistently, overexpression of ACE in the brain resident microglia, peripheral myelomonocytes, and macrophages can alleviate the symptoms of the double-transgenic APPSWE/PS1ΔE9 (AD+) mice [52,53]. Thus, in the AD model system in mice, the ADE activity can reduce Aβ -induced pathologic problems. The efficacy of the ADE activity seems to be consistent with the clinical studies with increased ACE and reduced risk of AD.

ACE is also known to have broad specificity to substrates, including enkephalins, C-terminal extended proenkephalins, luteinizing hormone-releasing hormone, substance P and a protected chemotactic tripeptide [54], cholecystokinin-8, gastrin analogues [55], and kinins [56]. However, it is not clear how the ACE substrates are relevant to AD, and thus we do not discuss them further.

### 3.5. ACE as a Link to Aging (Life Extension Model Systems)

Previous studies have investigated the effects of ACE inhibitors and homologs of ACE in model species. In the nematode, *Caenorhabditis elegans*, the *acn-1* gene, a homolog of nematode ACE, was used to explore the relation between the use of ACE inhibitors and longevity [57]. The application of the ACE inhibitor, Captopril, reduces the *acn-1* activity, leading to life extension, increased stress resistance, and a delay in age-related degenerative changes measured by pharyngeal pumping. Further analysis indicated that the lifespan effects of Captopril are additive to other known life-extending interventions (and genes), including the insulin/IGF-1 pathway (*age-1* and *daf-2*), caloric restriction (*eat-2*), a nicotinamide adenine dinucleotide (NAD)-dependent deacetylase (*sir-2.1*), heat shock (*hsf-1*), and mitochondria insufficiency (*isp-1*).

In the fruit fly, Drosophila melanogaster, another ACE inhibitor, Lisinopril, was used to study the impacts of Ance, an ortholog of ACE, in the three genotypes from the reference panel lines [58]. Mean lifespan was increased following the application of Lisinopril in three inbred lines from a natural population (DGRP73, CGRP229, and DGRP304). The effect of lisinopril showed a genotype-specific manner with the degree of change to lifespan (the highest effect in DGRP304), age-specific speed (the highest effect in DGRP229), endurance (the highest effect in DGRP229), and strength (the highest effect in DGRP229). The inbred lines that had an improvement of physical performance while on Lisinopril had a reduction in the age-related aggregation of protein in skeletal muscle, suggesting a role of stress resistance in lifespan extension. The results showed a significant involvement of skeletal muscle Ance in the lifespan of Drosophila species and that there is genetic variation in the phenotypic responses to ACE inhibitors. Mouse models have also been used to study the effect of ACE inhibitors on lifespan. A study that used the combined application of statin, simvastatin, and ACE inhibitor, Ramipril, resulted in an increased mean lifespan for a group of isocalorically fed mice subjects [59].

### 3.6. ACE as a Link to Stress Resistance and the Middle-Life Crisis Theory on Aging

ACE inhibitors and mutations are associated with life extension and stress resistance (discussed above). Increased resistance to multiple forms of stresses has been shown as a component of life extension in the model species, including yeasts, nematodes, fruit flies, and mice [60,61,62,63,64,65,66,67]. ACE as an angiotensin-converting enzyme has been used as a drug target to control hypertension and related complications. In contrast, ACE as an amyloid-degrading enzyme has been used to control more advanced phase accumulation of beta-amyloid. The different activities of ACE support the notion of aging stages from the transition state to the more advanced pathological state [68,69,70]. Furthermore, those are consistent with a previous study that indicates the underlying mechanisms of the human AD genes, including lipid metabolism, amyloid-related pathways, and neural and immune systems [10]. We see that age-related comorbidities occur during the middle of lifespan when the onset of hypertension and associated health conditions occur. At the stage of the lifespan, the ACE function as an angiotensin-converting enzyme is beneficial to the health condition, while the function as an amyloid-degrading enzyme is more important when an abnormal accumulation of beta-amyloid starts. ACE provides an example for the middle-life crisis theory on aging in which middle-life events result in aging and age-related diseases in late life. In the theory, the middle-life events explain the transition to aging from normal to more advanced age-related changes [69,70].

## 4. Conclusions and Perspective

Hypertension is major comorbidity that develops over time. First-line medications include ACE inhibitors that have been well established in medicine. In contrast, growing evidence suggests that ACE genetic variants are risk factors for AD and other neurological diseases. This seemingly controversial finding has been an intriguing topic but has never been discussed and clarified. To this end, we dissected the functions of ACE and explained this seemingly controversial finding. ACE inhibitors are beneficial to reduce hypertension and associated health conditions. Later in life, ACE as an amyloid-degrading enzyme starts to play a role in fighting against AD pathology, which is supported by clinical studies with increased ACE and reduced risk of AD (see Section 3.3). This later-life ACE function remains to be explored in humans, since it could provide a promising target for the treatment of AD and other related health conditions. The current understanding is that hypertension medication should be used to prevent vascular, stroke, and mixed dementia [71], while the treatment for AD-specific symptoms requires considerations. Although it has been suggested that ACE inhibitors may reduce the amyloid-degrading activity of ACE [39], ACE inhibitors can inactivate abnormal ACE/angiotensin II signaling that causes Aβ-induced neurodegeneration (Section 3.4). ACE inhibitors have been shown to reduce the symptoms of the AD mouse model [43,46]. Importantly, inhibition of ACE can extend lifespans in model systems, including the nematode, the fruit fly, and the mouse, suggesting potential health benefits during lifespans. Thus, we reason that ACE inhibitors may be beneficial until the accumulation of amyloids causes health problems. The areas of ACE inhibitors and ACE receptor inhibitors are promising fields for further exploration. Taken together, the current status of understanding ACE in medications still has open questions specific to hypertension, other age-related comorbidities, and types of dementia.

## Data Availability

Not applicable.
